# Adsorption properties of silica aerogel-based materials

**DOI:** 10.1039/d3ra02462h

**Published:** 2023-06-16

**Authors:** Kristina Goryunova, Yunis Gahramanli, Rena Gurbanova

**Affiliations:** a Geotechnological Problems of Oil, Gas and Chemistry Research Institute Baku City Azerbaijan kristina.goryunova@hotmail.com; b Chemistry and Inorganic Substances Technology Department, Azerbaijan State Oil and Industry University Baku City Azerbaijan

## Abstract

Silica aerogels have piqued the interest of both scientists and industry in recent decades due to their unusual properties such as low density, high porosity, low thermal and acoustic conductivity, high optical transparency, and strong sorption activity. Aerogels may be created *via* two-step sol–gel synthesis from different organosilicon compounds known as precursors. Various drying processes are employed to remove the solvent from the gel pores, the most common of which is the supracritical drying method. This paper highlights the potential of silica aerogels and their modifications as adsorbents for environmental cleanup based on recent researches. Following an introduction of the characteristics of aerogels, production techniques, and different categorization possibilities, the study is organized around their potential use as adsorbents.

## Abstract 2

This paper highlights the potential of silica aerogels and their modifications as adsorbents for environmental cleanup based on recent research.

## Graphical abstract

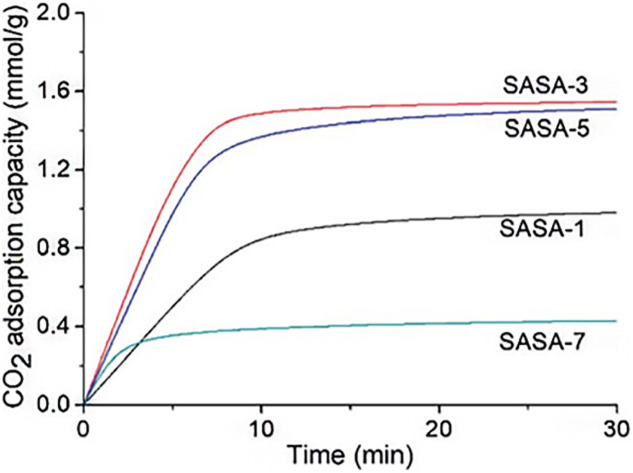

## 1. Introduction

Aerogels are solid, very porous materials with unique characteristics such as transparency, low density, large specific surface area, low heat and acoustic conduction, exceptional mechanical endurance, and high sorption activity.^1^ The majority of the described attributes attracted experts from a variety of scientific and technological fields.^2^ Dr Samuel Steven Kistler used the term “aerogel” in 1932 to describe a gel in which the liquid part was replaced by gas, but the silica networks remained intact.^3^ Kistler used a novel supercritical drying procedure^4^ in which the impregnating liquid was drained after it had been converted to a supercritical fluid. In his groundbreaking work, Kistler synthesized a wide range of aerogels. He was successful in producing mechanically weak alumina aerogels. To make the silica gels, sodium silicate was mixed with water and dried in supercritical ethanol.^1^ Originally, the chemical compounds known as precursors that contained the cations used to create an oxide gel were simply metallic salts. Nevertheless, dialysis was necessary to remove the sodium chloride generated in the silica gels created from Na_2_SiO_3_, followed by an exchange of water for a fluid such as ethanol. As a result, the alkoxides M(OR)_*n*_, a kind of chemical initially utilized by Ebelmen to synthesis silica aerogels,^5^ are the most preferred precursors. Peri was the first to use tetraethyl orthosilicate Si(OEt)_4_ as an aerogel precursor.^6^ The Teichner group^7,8^ concentrated on more substantial works in this area, as well as a wide spectrum of previously stated compositions. Low-temperature supercritical drying in CO_2_ is now distinguished from high-temperature supercritical drying in alcohol. These parameters, according to Pajonk,^9^ must be regulated during the process in order to prevent differential tensions. Apart from alkoxides, several more precursors are now accessible, including precursor molecules with an organic group as the ligand. Livage *et al.*^10^ presented a partial charge model for cations other than Si. As a result, the hydrolysis process with water that replaces OR ligands with OH ligands were quicker with alkoxides than with Si alkoxides. Sol–gel chemistry advancements, as outlined by Land *et al.*,^11^ enabled the manufacturing of “ambigel” type aerogels dried under ambient pressure settings. To reduce capillary drying stresses, organic compounds might be introduced to the liquid that was gelated in the simplest way. Several of these additions are classified as “Drying Control Chemical Additives” including glycerol, formamide, dimethyl formamide, and oxalic acid (DCCA).^12,13^ To collect comet dust in orbit, the Jet Propulsion Laboratory used the gradual absorption of shock energy by silica aerogel monoliths.^14^ This technique may also be used to gather aerosol particles,^15^ protect space reflectors, and build tank baffles.^16^ Aerogel particles can be utilized to give hardness, wear resistance, and thickening qualities in paints, varnishes, and tire elastomers.^17^ They may also be utilized to adsorb or extract chemical components in applications like as waste water purification, radioactive waste reduction,^16^ gas filtration,^18^ and heat storage systems.^19^ The optical transparency of silica aerogels was examined for application in optical devices, and it was revealed that the so-called two-step sol–gel synthesis, acid-catalyzed hydrolysis followed by base-catalyzed condensation, was particularly efficient.^20^ The Teichner group previously established that the refraction index could be changed between 1.01 and 1.03, making it perfect for use in Cerenkov counters.^21^ Because of their tunable pore size and high specific pore volume, silica aerogels are ideal for releasing pharmaceuticals or agricultural chemicals.^22^ Aerogels, on the other hand, can be used to adsorb or extract certain chemical compounds, such as waste water, radioactive waste confinement, or gas filtering. Reynes *et al.*^23^ suggested silica aerogels for long-term actinide waste storage since they are chemically stable with time on stream and have a very large relative pore volume. Another option for water sorbents for low-temperature heat storage is silica aerogels impregnated with CaCl_2_, LiBr, and MgCl_2_ salts. Its energy storage capacity *E* measured by differential scanning calorimetry (DSC) can reach 4.0 kJ g^−1^, which is a significant improvement over that of traditional sorbents like zeolites and unimpregnated silica gels.^19^

## 2. Classification and properties

**Fig. 1:**
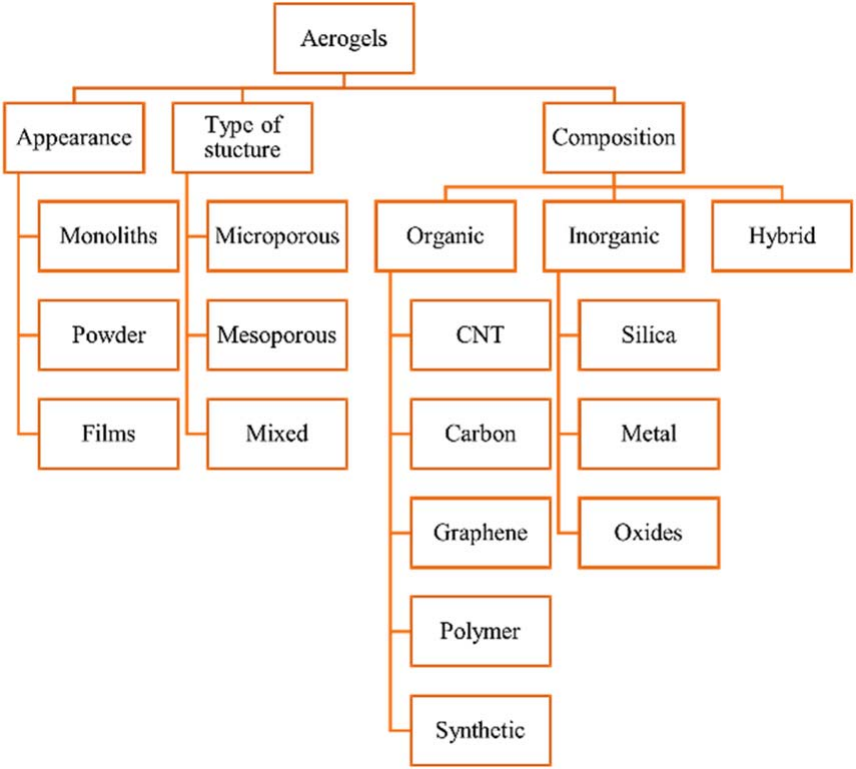
Classification of aerogels.

The appearance (monoliths, films, and powder) structure (mesoporous, microporous, and mixed porous) and composition of aerogels are used to classify them. Aerogels can be organic, inorganic, or hybrid (Fig. 1).

### 2.1. Physical properties

The part of inorganic chemistry researches has focused its attention on silica aerogels and their applications. Due to their high specific surface area (500–1000 m^2^ g^−1^), low density (0–003 g cm^−3^), and nanoporous structure (90–98%), silica aerogels exhibit exceptional properties.^24^ With a thermal conductivity of 3 mW m^−1^ K^−1^ and an acoustic velocity of 100 m s^−1^, silica aerogels are well-known for being thermal and acoustic insulators.^25^Table 1 demonstrates typical silica aerogel properties.

**Table 1:** Typical properties of silica aerogel

Property	Value
Apparent density	0.003–0.35 g cm^−3^
Internal surface area	500–1000 m^2^ g^−1^
Mean pore diameter	∼20 nm
Primary particle diameter	2–5 nm
Refractive index	1.0–1.08
Coefficient of thermal expansion	2.0–3.0 × 10^−6^
Dielectric constant	∼1.1
Sound velocity	100 m S^−1^

#### 2.1.1. Thermal conductivity

Due to their extremely low heat conductivity (0.01 W m^−1^ K^−1^ at ambient pressure), silica aerogels are the most well-known thermal insulating material.^26^ In addition, silica aerogels can be made optically transparent but are very fragile. They thus provide a variety of applications for opaque or transparent insulating components.^27,28^ The aerospace and aviation industries are increasingly using silica aerogel as a thermal insulator.^29^ During the most recent PATHFINDER MARS mission, they were used to insulate the Sojourner Mars Rover. Aerogels have been studied for use in a mission similar to that of the European Retrieval Carrier (EURECA) satellite.^30^

#### 2.1.2. Acoustic properties

Silica aerogels' ability to insulate heat from the environment is inextricably linked to its acoustic qualities. Because heating enhances the degree of connectedness of the silica nanoparticle pearl-necklace network structure, heat-treated aerogels have greater sound velocities than untreated aerogels.^31^ The type and pressure of the interstitial gas, the density of the aerogel, and, more generally, the texture, all affect acoustic transmission in aerogels.^32^ Aerogels made of silica are excellent at isolating sound. An acoustic wave's amplitude and velocity decrease as wave energy is gradually transferred from the gas to the solid network of the aerogel.^33–35^

#### 2.1.3. Dielectric properties

Silica aerogels have an extremely low relative dielectric constant of 1.1.^36^ Thin film silica aerogels have the potential to be and are currently being studied as extremely low dielectric constant materials for computer integrated circuits. By modifying the surface of silica aerogel, excellent electret materials can also be produced.^37^

#### 2.1.4. Optical properties

When a transparent thermal insulation, such as windows, is required, silica aerogels' optical transmission and scattering properties are a crucial component. These properties are frequently combined with their thermal properties. The initial analysis of this subject was provided by Pajonk.^38^ The optical quality of silica aerogels is diminished despite their high transparency and visible light transmittance.^39^ All silica aerogels scatter some of the transmitted light. It was found that a two-step gelation catalysis method produced excellent transparency outcomes. Tetramethyl orthosilicate (TMOS) aerogels can frequently reach optical transmittance ratios of up to 93 percent at 900 nm when they are made in methanol.^40,41^ Aerogels are suitable materials for use in Cerenkov counter radiators as a result.^42^

## 3. Synthesis

The production of silica aerogels may be separated into four major processes:^43^

(1) Sol formation. In a wide range of stable solutions, alkoxide or the solvated metal precursor (sol) is generated.

(2) Gelation process. Gelation is the consequence of a polycondensation or polyesterification process that raises the viscosity of the solution and generates an oxide- or alcohol-bridged network (gel).

(3) Aging of the gels. Aging of the gel (syneresis), which is followed by contraction of the gel network and solvent evacuation from gel pores until the gel converts into a solid mass. The gel grows stronger as it ages, reducing shrinking after drying.

(4) Drying of the gels. At this phase, the gel should be cleansed of pore liquid. Drying occurs under certain conditions to prevent the gel structure from collapsing.

**Fig. 2:**
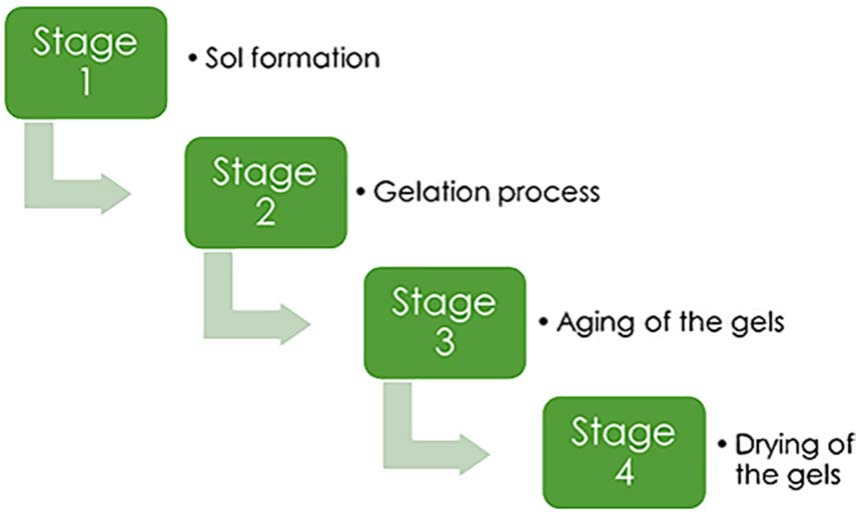
Main stages of aerogel synthesis, Gahramanli *et al.*, ISPC KOGF-2022.^43^

These four basic processes (Fig. 2) are used in all techniques of making aerogel. Other treatments can also have an impact on the final product's structure.

### 3.1. Sol formation and gelation processes

Sol–gel processing is a popular and dependable approach for manufacturing materials, particularly metal oxides with homogeneous, tiny particle sizes and a wide range of morphologies.^44^ To do this, a system must transition from a liquid “sol” phase to a solid “gel” phase. The sol–gel process has three stages: sol creation, gelation, and gel aging.

In contrast to crystallization from solution or the synthesis of an inorganic network by a chemical reaction in solution at low temperature, sol–gel processing refers to the production of an amorphous network.

Precursors are soluble starting ingredients for the sol–gel process that must be sufficiently reactive to participate in the gel formation process.^46^ Alkoxides are the most common sol–gel precursor, since they are commonly available. The most popular precursors are tetramethyl orthosilicate (TMOS) and tetraethyl orthosilicate (TEOS), which were first used in aerogel synthesis as precursors by Ebelmen.^5^ Currently, there is a wide range of alkoxide-derived precursors used, which are mentioned in Table 2.

**Table 2:** List of alkoxide-derived precursors and researches established on them

Precursor	Research
Polyethoxydisiloxane (PEDS)	Einarsrud *et al.*,^47^ Deng *et al.*^48^
Methyltrimethoxysilane (MTMS)	Venkastewara *et al.*,^49^ Rassy *et al.*^50^
Methyltriethoxysilane (MTES)	Harreld *et al.*^51^
3-(2-Aminoethylamino)propyltrimethoxysilane (EDAS)	Allié *et al.*^52^
Noctyltriethoxysilane	Rodriguez *et al.*^53^
Dimethyldiethoxysilane	Venkateswara *et al.*^54^
Perfluoroalkysilane (PFAS)	Zhou *et al.*^55^
*N*-isopropylacrylamide	Mattea *et al.*^56^
Polymethylsilsesquioxane	Wu *et al.*^57^

According to Gurav *et al.*,^45^ a variety of factors, including the activity of the metal alkoxide, the water/alkoxide ratio, the pH and temperature of the solution, the kind of solvent employed, and any additions, may influence the hydrolysis and condensation processes. A depiction of a nanostructured approach that links Si atoms given by precursor molecules *via* siloxane bridges (≡Si–O–Si≡). In organic chemistry, these alterations are analogous to polymerization. At the first phase of the sol–gel process, dispersed solid colloidal silica particles or linear oligomers are formed. In the second phase, while still in the solvent, these basic particles can link together to create a three-dimensional open network structure known as a gel, with the container acting as the only constraint. Gelation is the continuous transformation of a sol into a gel.

Hydrolysis:

1 ≡Si–OR + H_2_O ↔ ≡Si–OH + R–OH

Polycondensation:

2 ≡Si–OH + HO–Si≡ ↔ ≡Si–O – Si≡ + H_2_O

Both of these processes require a catalyst, for instance, acid catalyst for hydrolysis process (HCl, H_2_SO_4_, HF, HNO_3_, oxalic acid, formic acid), and base catalyst for polycondensation process (NH_4_F, NH_4_Cl, NH_4_OH). The sol preparation is very different for waterglass-based aerogels. Utilizing ion exchanging techniques or ion exchanging resins (strong acidic cation resin, sulphonated polystyrene type), the Na ions from sodium silicate must be removed. The pH of sodium silicate solution is 12, but after ion exchange, the resulting sol has a pH of 2. The resulting wet gel was then aged for three hours at 60 °C using an NH_4_OH solution as the base catalyst. In line with Lee *et al.*, the density and porosity of aerogel can be changed by raising the pH of the sol.^58^

### 3.2. Ageing of the gels

**Fig. 3:**
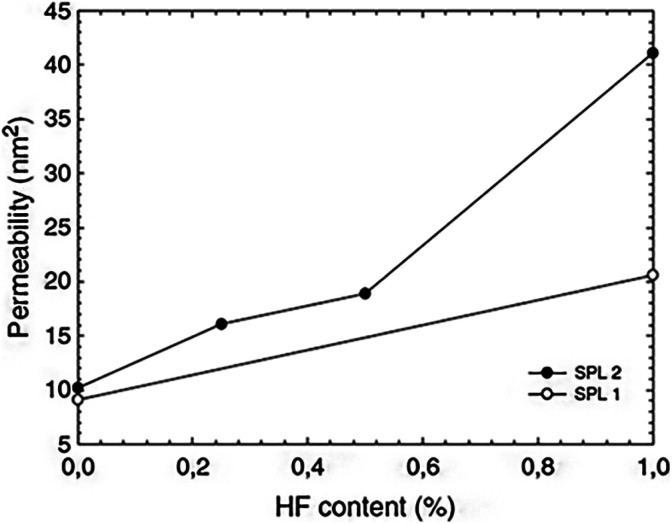
Absorption coefficient of silica wet gels (before drying) as a function of HF concentration in an aging bath containing 3 vol% water and consisting of ethanol (SPL1, white spots) or ethylacetoacetate (SPL2, solid marks) Strøm *et al.*, *J. Sol-Gel Sci. Technol.*, 2007.^59,61^

Numerous different procedures are utilized to age silica gels before drying. The goal of this stage is to strengthen the robust skeleton in order to prevent breakage during the drying process. Aging is another term for the syneresis process, which involves changing the content of the liquid component trapped in the pores of the gel. Additional condensation and precipitation processes might exist. The kinetics are affected by the pH and type of solvent. Recent research has shown that there are effective aging alternatives that simultaneously improve permeability and mechanical properties. They might involve adding larger precursor molecules like polyethoxydisiloxanes,^47^ for instance, or they might just involve adding a diluted HF solution without any additional silica precursor^59^ (Fig. 3). It has also been demonstrated that controlling the temperature and simply aging the wet gel thermally in water can be a crucial step in reducing the gel's microporosity before drying.^60^

### 3.3. Drying of the gels

Drying of the gel is the final and most crucial stage in the production of aerogels. There are three main routes used for this process:

(1) Evaporation – crossing the liquid–gas equilibrium curve.

(2) Freeze–drying – bypass the triple point.

(3) Supercritical drying – bypass the critical point.

#### 3.3.1. Ambient pressure evaporation

Ambient pressure drying is one of the most intriguing low-cost drying techniques for aerogels. There are typically two phases to this strategy. To stop water adsorption by creating hydrophobic aerogels, all OH groups must first be sylated. This is accomplished by substituting an anhydrous solvent for the current one and adding a sylation agent, such as hexamethyldisilazane (HMDS).^63^ Second, three phases of evaporation are used to dry the material at standard pressure. The first phase of drying begins during a heating interval, when the volume loss of the gel balances out the volume loss of the evaporated liquid as free water continues to migrate to the outer surface due to capillary forces. The diffusive motion of vapor predominates during the second stage of drying, also known as the deceleration period, allowing the liquid to slowly escape outwards. After the surface has dried to the point that hydraulic conductivity is almost negligible, the third stage of evaporation begins. Evaporation is decreased to a small fraction of the rate recorded when the surface is moist during this low-rate stage.^65^

#### 3.3.2. Freeze–drying

In this drying process, there is no distinction between the liquid and gaseous phases, so capillary pressure is minimal. In this case, the solvent should be switched out in favour of one with a low coefficient of expansion and high-pressure sublimation, followed by freezing and vacuum sublimation of the pore liquid.^62^ Acquired a substance called cryogel. Because cryogels can only be purchased as powders, this technology has one major drawback.

#### 3.3.3. Supercritical drying

**Fig. 4:**
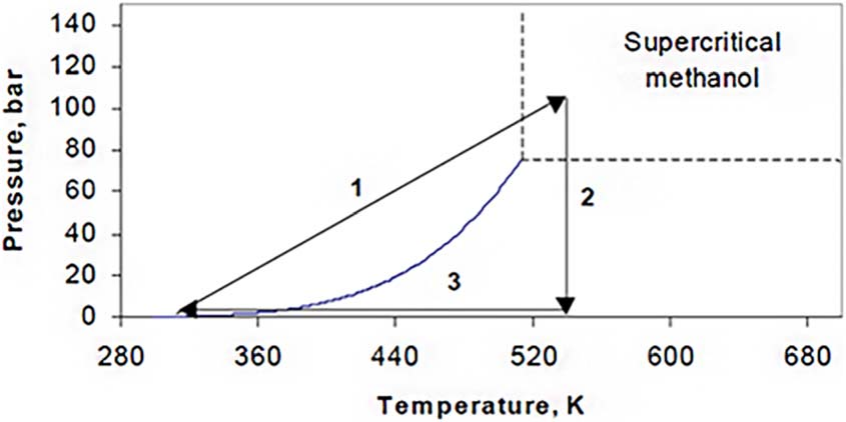
High temperature drying conditions (for methanol).

Supercritical drying can be done in two different ways: high-temperature supercritical drying (HTSCD) and low-temperature supercritical drying (LTSCD). HTSCD was used by Kistler,^4^ and it is still frequently used to make silica aerogels today. The procedure is finished with enough methanol as the solvent, and the wet gel is autoclaved. Pressure rises as temperature gradually rises. In order to achieve values above the solvent's critical points (Fig. 4), both temperatures are adjusted.

**Fig. 5:**
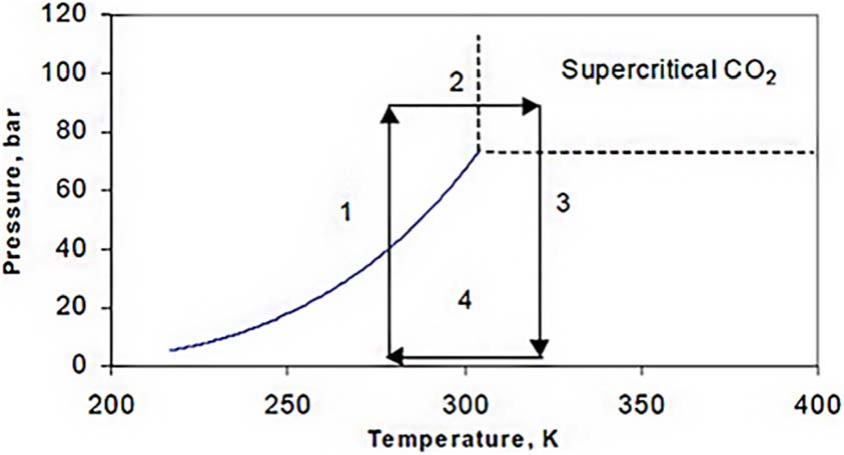
Low temperature supercritical drying conditions (for liquid CO_2_).

However, due to the combination of high temperatures and high pressures, as well as the flammability of solvents, this method can present problems. Depending on the liquid used to impregnate the wet gel, as shown in Table 3, the fundamental requirements vary significantly (Fig. 5).

**Table 3:** Critical point conditions of different fluids^64^

Fluid	Formula	*T*_c_ (°C)	*P*_c_ (MPa)
Water	H_2_O	374.1	22.04
Ethanol	C_2_H_5_OH	243.0	6.38
Methanol	CH_3_OH	239.4	8.09
Carbon dioxide	CO_2_	31.0	7.37

**Fig. 6:**
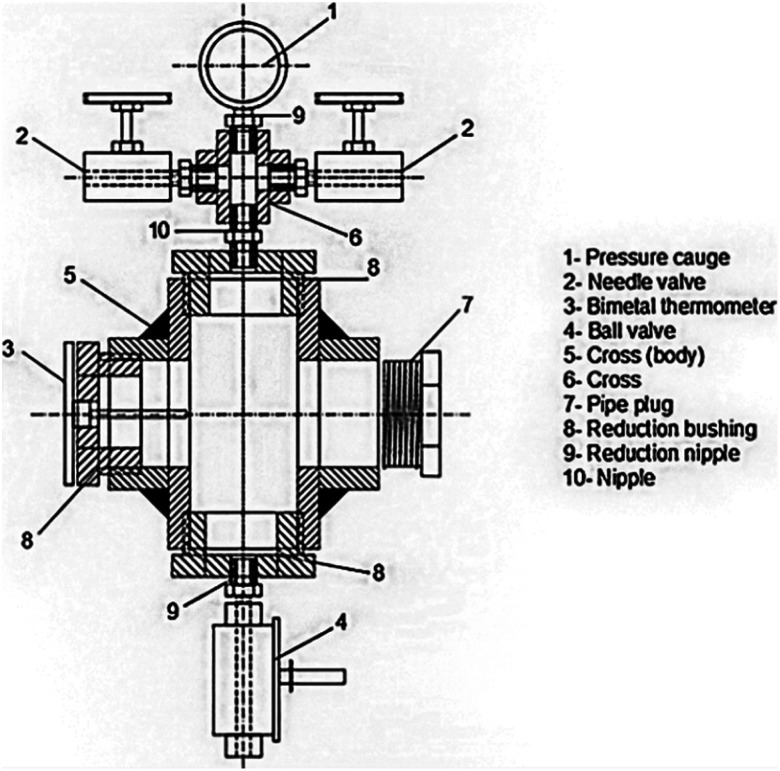
Schematic representation of supercritical autoclave, Gahramanli *et al.*, ISPC KOGF-2022.^43^

For drying aerogels, Tewari *et al.*^66^ suggested a different technique. Before drying, a liquid with a critical point close to room temperature replaces the gel's solvent. The most practical solution turned out to be liquid CO_2_. The fact that LTSCD can be carried out at low temperatures (40 °C) and moderate pressures (8.09 MPa) is beneficial (Fig. 6).

## 4. Silica aerogel-based adsorbents

### 4.1. Oil adsorption with aerogel

**Fig. 7:**
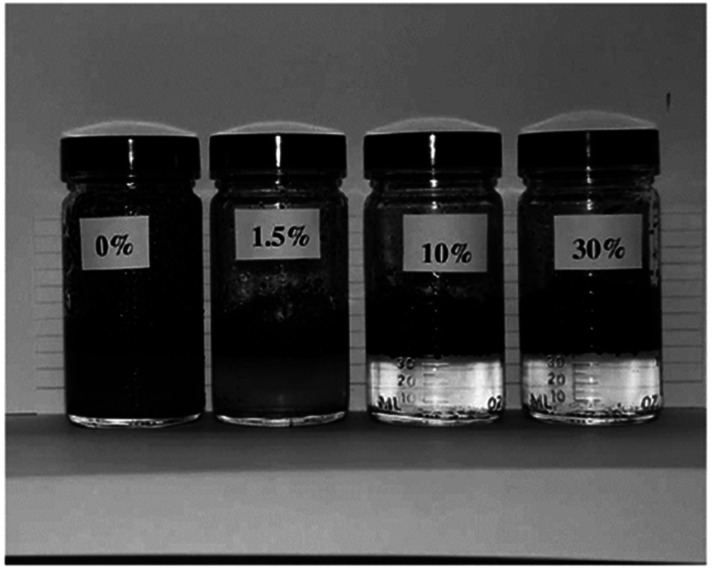
30%, 1.5% and 0% CF_3_-aerogels mixed with Prudhoe Bay crude oil with 3 wt% NaCl Reynold *et al.*, *J. Non-Cryst. Solids*, 2001.^67^

Silica aerogel has not been used extensively for the adsorption of crude oil from water. In the research of Reynolds *et al.*^67^ Crude oil was removed from an oil and salt-water mixture using powdered CF_3_-functionalized aerogels. Using TMOS as a precursor, CF_3_(CH_2_)_2_Si(OCH_3_)_3_ as a functionalizing agent, methanol as a solvent, and diluted NH_4_OH solution as a base catalyst, the aerogel had been made using the sol–gel method. In order to produce hydrophobic aerogels, supercritical drying has been found to be the most effective method, due to reason that heating in air caused decomposition of the gel. The oil in the water was entirely removed by aerogels, regardless of the functionalized agent's concentration (percentage of CF_3_(CH_2_)_2_-group) (Fig. 7). According to this, there could be up to 237 parts oil to one part aerogel. Exfoliated graphite, for instance, can absorb 80 times its weight in oil, making it one of the best oil-absorbing materials.^68^

### 4.2. Use of aerogels for the elimination of VOC

**Fig. 8:**
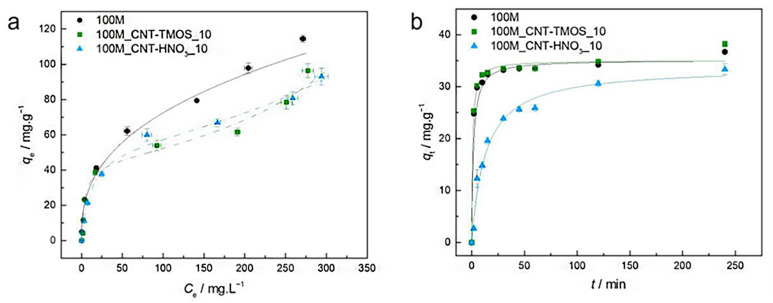
(a) Experimental equilibrium data and the best fitted isotherm model for adsorption of benzene into the studied aerogels (solid line – Freundlich; dashed line – BET). (b) Representative plots of the fit of nonlinearized form of pseudo-second order equation (solid line) to the data of experimental adsorption capacity as function of time, for the studied adsorbents towards benzene (*C*_0_ = 100 ppm, 15 rpm, 20 °C) Lamy-Mendes *et al.*, MDPI Toxics, 2023.^71^

**Fig. 9:**
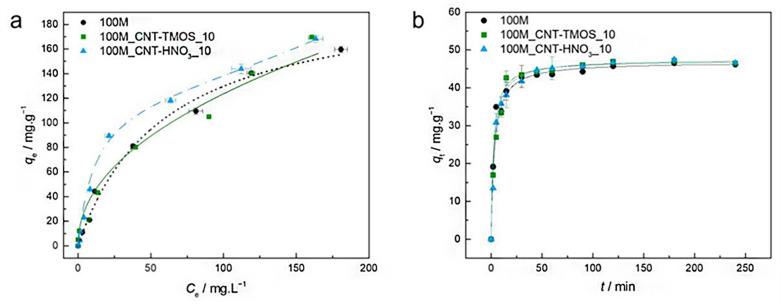
(a) Experimental equilibrium data and the best fitted isotherm model for adsorption of toluene into the studied aerogels (dotted line – Langmuir; solid line – Freundlich; dashed line – BET). (b) Representative plots of the fit of non-linearized form of pseudo-second order equation (solid line) to the data of experimental adsorption capacity as function of time, for the studied adsorbents towards toluene (*C*_0_ = 100 ppm, 15 rpm, 20 °C) Lamy-Mendes *et al.*, *MDPI Toxics*, 2023.^71^

The most popular method for removing VOCs from the atmosphere is adsorption. According to Fairén-Jiménez and colleagues,^69^ aerogels can easily satisfy the adsorption requirements, and their use in the removal of the toxic pollutants mentioned above is very promising. With a saturation adsorption capacity of 3000 mg g^−1^, silica aerogel was identified as a promising sorbent to bind benzene vapour, whereas activated carbon powder had a 500 mg g^−1^ adsorption capacity.^70^ According to recent research of Lamy-Mendes *et al.* (2023),^71^ non-polar chemical molecules such as benzene, toluene, and xylene may be successfully removed using MTMS-based aerogels. In the presence of carbon nanotubes, removal rates of more than 70% were recorded for toluene concentrations as high as 400 mg L^−1^ (Fig. 8). Similar results were obtained with xylene. Removal efficiency more than 70% were obtained, but removal efficiencies for NPX were consistently better than 94% for the composites containing amino groups (Fig. 9).

### 4.3. Adsorption of various components from air using aerogels

**Fig. 10:**
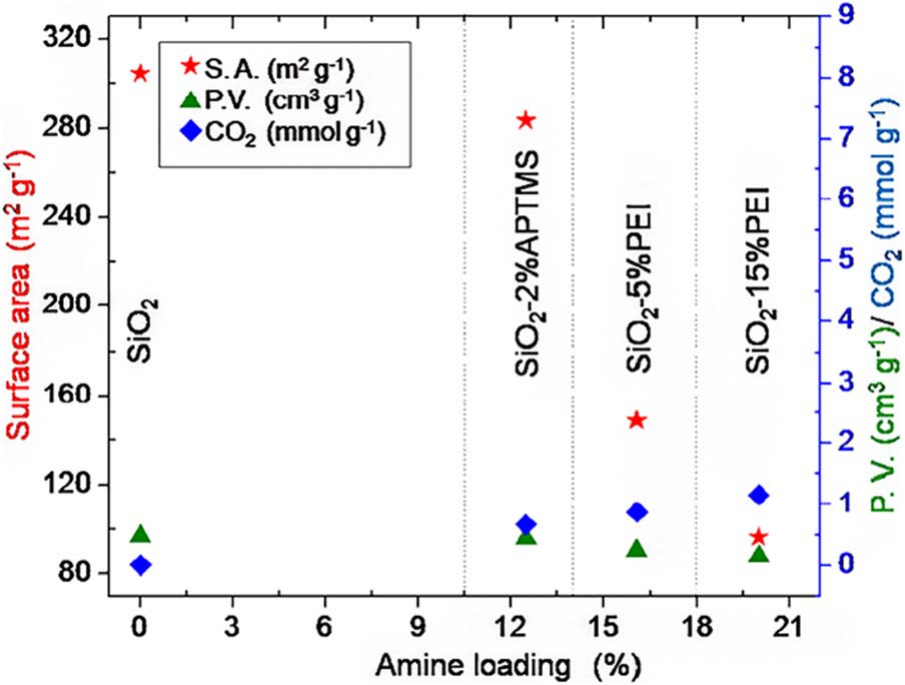
Surface properties and CO_2_ adsorption as a function of amine loading, Minju *et al.*, *Chem. Eng. J.*, 2015.^73^

According to the research of Wilson *et al.* (2020),^72^ TMOS-based silica aerogel, TMS-modified silica aerogel and silica gel adsorbed CO_2_, N_2_, O_2_ and Ar from air (Fig. 12). Regarding to the impact of changing surface of the silica aerogel and the pore size also changes the adsorption of different components of air. TMOS-based silica aerogel and TMS-modified silica aerogel showed the best result than pure silica gel on adsorption of CO_2_ gas from air. In the study of Minju *et al.* (2015),^73^ waterglass-based silica aerogels modified with PEI and APTMS successfully adsorbed CO_2_ at low temperature. The amine modification decreased bulk surface area and gel support resulted enhanced CO_2_ adsorption (Fig. 10).

**Fig. 11:**
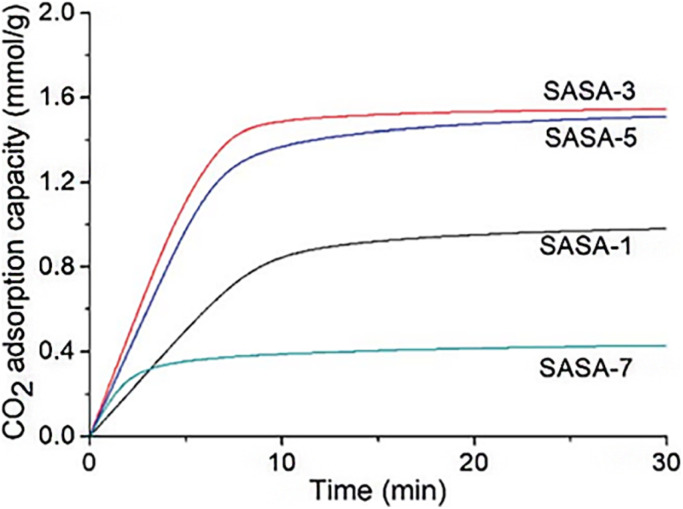
CO_2_ adsorption kinetics of the SASA samples with dry 1% CO_2_ at 25 °C, Xing *et al.*, *RSC Adv.*, 2020.^74^

Xing *et al.* conducted additional research in this area. 3-Aminopropyltriethoxysilane was grafted onto the silica gel framework in the study from (2020)^74^ by the research team. The spherical amine grafted silica gels were vacuum dried to create the spherical amine grafted silica aerogels (SASA). The CO_2_ adsorption capacity was dependent on the amine surface content and adsorption temperature, as was mentioned in earlier research.^73^ With dry 1 percent CO_2_, the SASA has a higher CO_2_ adsorption capacity than its cutting-edge competitors. It suggests that this work can offer an affordable and environmentally friendly method for designing a useful and regenerable adsorbent material for low-concentration CO_2_ capture. The SASA can be used in a fluidized bed reactor without pelleting, which is required for powder-like adsorbents. CO_2_ adsorption performances of the SASA in the fluidized bed reactor are currently being studying (Fig. 11).

### 4.4. Dye adsorption using silica aerogels

Meng *et al.*^75^ for the purpose of removing dye from wastewaters prepared hollow silica aerogel (SA) fibers that were engineered based on a wet-spinning method. Both phenyltrimethoxysilane (PTMS) and 3-aminopropyltrimethoxysilane (APTMS) were used to modify the surfaces. The authors also added photocatalysis-active nanoparticles to SA fibers that were used for commercial purposes. The fastest adsorption for CR and removal percentage for those fibers obtained using APTMS as the surface modifier were 86.3 percent after 5 minutes, while 98.2 percent for those fibers obtained using non-surface-modified SA. Last but not least, PTMS-modified SA fibers demonstrated a high removal speed for both CR and MB. In other study, Najafidoust *et al.* in order to combine the benefits of the distinctive layered structure of bismuth oxyhalides (BiOX, where X = Br, Cl, and I) and the large surface area of silica aerogels,^76^ synthesized a BiOI/SA using a sono-solvothermal method. Due to its small bandgap (*E*_g_ = 1.7–1.9 eV), BiOI is the type of BiOX that is most frequently used as a photocatalyst. As examples of wastewater pollutants, three organic dyes—MB, AO7, and RhB—were used. Under solar light, the BiOI/SA photocatalyst's catalytic performance was evaluated, and removal rates of MB, RhB, and AO7 of 92.11%, 65.4%, and 22.31% in 120 minutes, respectively, were found (Fig. 13). The influence of the initial dye solution's pH on the removal of MB was assessed, and it was discovered that a pH of 9 was ideal, leading to a removal of 96.5 percent.

**Fig. 12:**
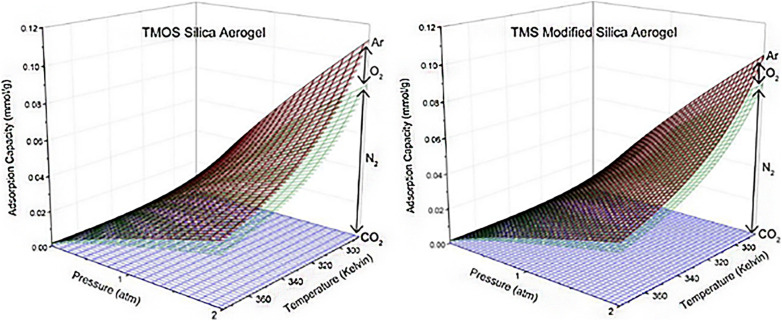
Cumulative adsorption capacities for TMOS silica aerogel and TMS-modified silica aerogel of CO_2_, N_2_, O_2_, and Ar at ambient air concentrations of 400, 780 840, 209 460, and 9300 ppm, respectively, from temperatures of 285 K–375 K and pressures of 0–2 atm. These concentrations correspond to those found in typical ambient air, Wilson *et al.*, *Microporous Mesoporous Mater.*, 2020.^72^

### 4.5. Removal of heavy metals using silica aerogels

Vareda *et al.* (2019)^77^ adsorbed a number of heavy metals that are present in groundwater and waterways. The aerogel-like materials made of silica were given mercapto or amine-mercapto group functionalizations. As precursors, TEOS, MTES, and MPTMS were used to create the mercapto-functionalized aerogels, while APTMS was also used to create the amine-mercapto-functionalized aerogels. A 5 day aging period was followed by either oven drying to produce xerogels or supercritical CO_2_ drying to produce aerogels. The percentages of the metals that were removed were 40 percent for zinc, 39-point one percent for cadmium, 38-point five percent for nickel, and 39-point eight percent for chromium. Imamoglu *et al.*^78^ revealed that 3-(2-aminoethylamino)propyl bonded silica gel loaded columns may be used to preconcentrate Au(iii), Pd(ii), and Cu(ii). Trace quantities of gold(iii) and palladium(ii) in ore or environmental samples can be preconcentrated, separated, and evaluated by AAS utilizing an AEAP-SG column. Gold(iii) and palladium(ii) ions may be separated from copper(ii) ions using modified silica gel. It is straightforward to make 3-(2-aminoethylamino)propyl bound silica gel. At high flow rates and from large volumes of solution, the modified silica gel can adsorb gold(iii), palladium(ii), and copper(ii) ions (Fig. 14 ).

**Fig. 13:**
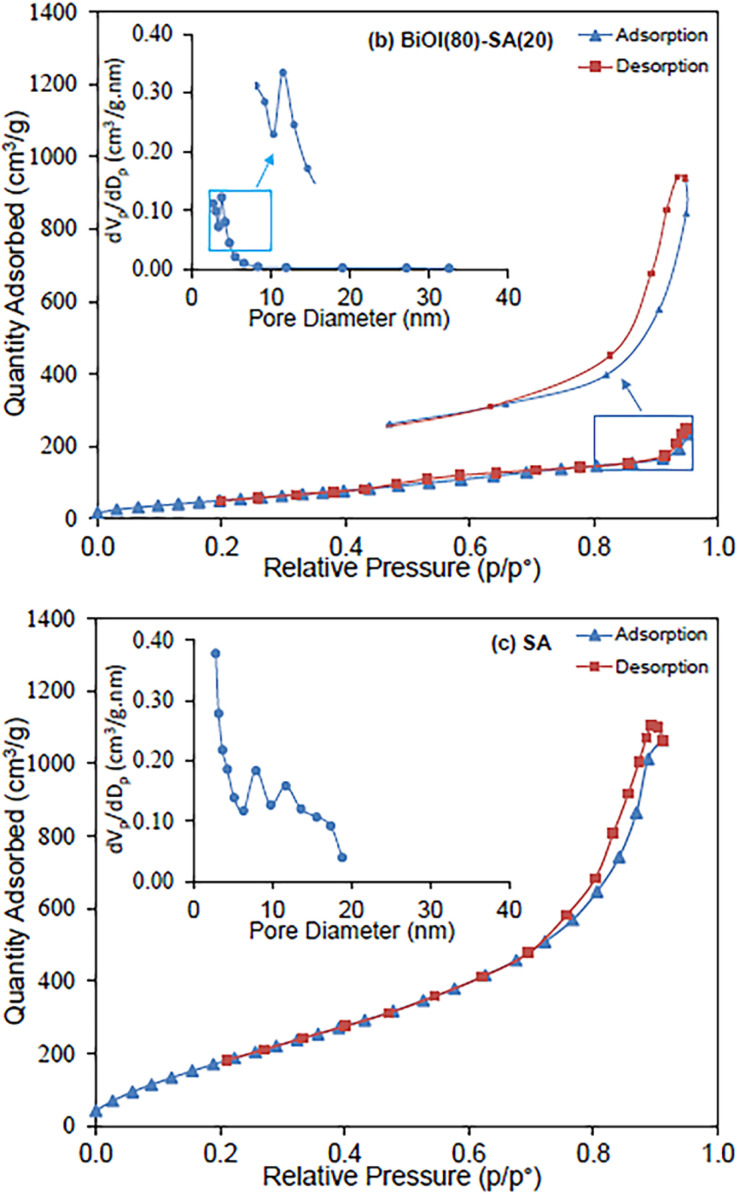
Adsorption/desorption isotherms and pore size distribution of nanostructured flowerlike BiOI photocatalyst over silica-aerogel: (a) BiOI, (b) BiOI(80)-SA(20) and (c) SA, Najafidoust *et al.*, *Sep. Purif. Technol.*, 2019.^76^

**Fig. 14:**
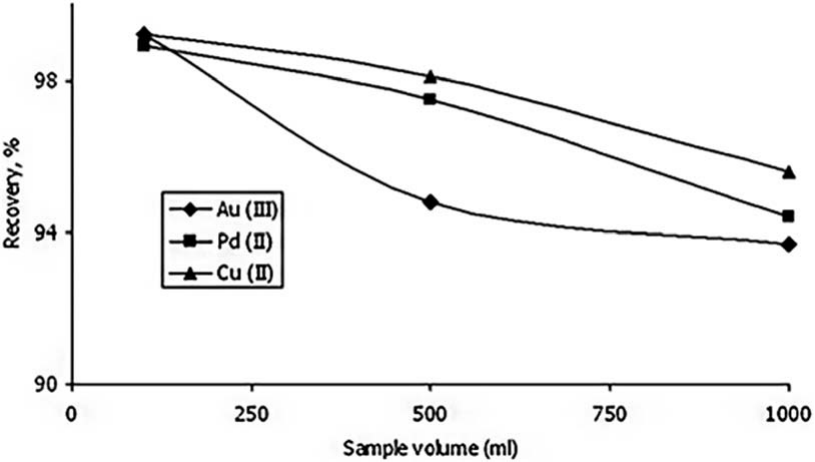
The effect of the solution volume on the recovery of gold(iii), palladium(ii) and copper(ii) (amount of each metal, 0.05 mg; eluent, 10 mL of 0.1 mol L^−1^ potassium cyanide; flow rate of the solution, 7 mL min^−1^; pH of gold(iii) solution 2.5; pH of palladium(ii) solution 1.0; pH of copper(ii) solution 5.5), Imamoglu *et al.*, *Cent. Eur. J. Chem.*, 2005.^78^

## 5. Conclusion

Silica aerogel-based materials have excellent properties such as low density, high porosity, and high specific surface area while being environmentally benign. These exceptional properties, along with the versatility of their wet synthesis method, have made these materials a potential environmental protection option. In most studies, silica aerogels and their modifications have been shown to be excellent sorbent materials with high sorption capacity towards the target components. The ability of silica aerogels to regenerate during the process is especially appealing from the aspect of economics and large-scale manufacture. Despite all of this, aerogels are still poorly understood and require more investigation in many fields of industry and science.

## Conflicts of interest

There are no conflicts to declare.

## Supplementary Material
